# Antimicrobial treatment for ventilator-associated tracheobronchitis: a randomized, controlled, multicenter study

**DOI:** 10.1186/cc6890

**Published:** 2008-05-02

**Authors:** Saad Nseir, Raphaël Favory, Elsa Jozefowicz, Franck Decamps, Florent Dewavrin, Guillaume Brunin, Christophe Di Pompeo, Daniel Mathieu, Alain Durocher, the VAT Study Group

**Affiliations:** 1 Réanimation Médicale, boulevard du Pr Leclercq, Hôpital Calmette, CHRU de Lille, 59037 Lille Cedex, France; 2 Laboratoire d'Evaluation Médicale, EA 2690, Université Lille II, 1 place de Verdun, 59045 Lille, France; 3 Centre d'Investigation Clinique, boulevard du Pr Leclercq Hôpital Cardiologique, CHRU de Lille, 59037 Lille Cedex, France; 4 Réanimation Neurochirurgicale, CHRU de Lille, Hôpital R. Salengro, CHRU de Lille, 59037 Lille Cedex, France; 5 Réanimation Polyvalente, Hôpital Régional, Avenue Désandrouin, BP 479, 59322 Valenciennes Cedex, France; 6 Réanimation Polyvalente, CH Duchenne, rue Jacques Monod, BP 609, 62321 Boulogne Sur Mer, France

## Abstract

**Introduction:**

Ventilator-associated tracheobronchitis (VAT) is associated with increased duration of mechanical ventilation. We hypothesized that, in patients with VAT, antibiotic treatment would be associated with reduced duration of mechanical ventilation.

**Methods:**

We conducted a prospective, randomized, controlled, unblinded, multicenter study. Patients were randomly assigned (1:1) to receive or not receive intravenous antibiotics for 8 days. Patients with ventilator-associated pneumonia (VAP) prior to VAT and those with severe immunosuppression were not eligible. The trial was stopped early because a planned interim analysis found a significant difference in intensive care unit (ICU) mortality.

**Results:**

Fifty-eight patients were randomly assigned. Patient characteristics were similar in the antibiotic (n = 22) and no antibiotic (n = 36) groups. *Pseudomonas aeruginosa *was identified in 32% of VAT episodes. Although no difference was found in mechanical ventilation duration and length of ICU stay, mechanical ventilation-free days were significantly higher (median [interquartile range], 12 [8 to 24] versus 2 [0 to 6] days, *P *< 0.001) in the antibiotic group than in the no antibiotic group. In addition, subsequent VAP (13% versus 47%, *P *= 0.011, odds ratio [OR] 0.17, 95% confidence interval [CI] 0.04 to 0.70) and ICU mortality (18% versus 47%, *P *= 0.047, OR 0.24, 95% CI 0.07 to 0.88) rates were significantly lower in the antibiotic group than in the no antibiotic group. Similar results were found after exclusion of patients with do-not-resuscitate orders and those randomly assigned to the no antibiotic group but who received antibiotics for infections other than VAT or subsequent VAP.

**Conclusion:**

In patients with VAT, antimicrobial treatment is associated with a greater number of days free of mechanical ventilation and lower rates of VAP and ICU mortality. However, antibiotic treatment has no significant impact on total duration of mechanical ventilation.

**Trial registration:**

ClinicalTrials.gov, number NCT00122057.

## Introduction

Ventilator-associated tracheobronchitis (VAT) is common among mechanically ventilated critically ill patients [1-3]. Previous studies found VAT to be associated with increased duration of mechanical ventilation and intensive care unit (ICU) stay [1,4,5]. VAT is probably an intermediate process between lower respiratory tract colonization and ventilator-associated pneumonia (VAP). Postmortem studies showed a continuum between bronchitis and pneumonia in mechanically ventilated ICU patients [6].

Few studies have evaluated the impact of antibiotic treatment on the outcome of critically ill patients with VAT [1,4,5]. In a prospective observational study [1], our group investigated the impact of antibiotic treatment on the outcome of patients with VAT. Among the 201 patients with VAT, 136 received antibiotics. The mortality rate was significantly lower in VAT patients who received antibiotics than in those who did not receive antibiotics. However, after exclusion of VAT patients who developed subsequent VAP, no significant difference was found in mortality rate. A beneficial effect of antimicrobial treatment on the duration of mechanical ventilation was also suggested by an observational case-control study performed in chronic obstructive pulmonary disease (COPD) patients with VAT [4]. However, another case-control study performed in VAT patients without chronic respiratory failure found no impact of antimicrobial treatment on the duration of mechanical ventilation [5]. Furthermore, it has been shown that systemic antibiotics have no effect on the transition from VAT to VAP [1].

Although no firm evidence on the beneficial effects of antibiotic treatment in patients with VAT exists, ICU physicians frequently treat these patients with antibiotics [7-10]. However, excessive usage of antibiotics in the ICU is associated with the subsequent emergence of multidrug-resistant (MDR) bacteria and worse outcome [11-14]. In their recent guidelines [15], the American Thoracic Society (ATS) and the Infectious Disease Society of America recommended the performance of randomized studies to determine whether patients with VAT should be treated with antibiotics. Therefore, we conducted this prospective, randomized, controlled, multicenter study to determine the impact of antimicrobial treatment on outcome in VAT patients.

## Materials and methods

The study was conducted in 12 ICUs in the north of France from June 2005 to June 2007. The study protocol was approved by the institutional review board on human research of the Lille university hospital. All patients or their next of kin gave written informed consent before enrolment in the study.

The eligibility criteria for the study were age older than 18 years and the presence of a first episode of VAT diagnosed more than 48 hours after starting mechanical ventilation. Before random assignment, patients were excluded if they (a) were pregnant, (b) had a history of severe immunosuppression, (c) had a tracheostomy at ICU admission (however, patients were eligible if they had a tracheostomy performed after ICU admission), (d) had a VAP before VAT, (e) had already participated in this study, (f) were already included in another trial, or (g) had little chance of survival as defined by a Simplified Acute Physiology Score (SAPS II) of greater than 65 points.

### Random assignment and antibiotic treatment

Patients were randomly assigned to receive or not receive intravenous antimicrobial treatment for 8 days. The duration of antimicrobial treatment was based on the results of a large multicenter randomized study on the duration of antibiotic therapy in patients with VAP [16]. A computer-generated random assignment list in balanced blocks of four was assigned to each participating ICU. Treatment assignments were contained in sealed opaque individual envelopes that were numbered sequentially.

The study was not blinded. The initial empirical antibiotic regimen was based on results of the last endotracheal aspirate culture. In the antibiotic group, the initial antibiotic treatment was modified, if inappropriate, after receipt of definitive microbiologic results identifying the pathogen(s) and its susceptibility patterns. In the no antibiotic group, antibiotics could be given for subsequent VAP or infections other than VAT or subsequent VAP.

### Study population

In all patients, quantitative endotracheal aspirate was performed at ICU admission and weekly thereafter. In addition, quantitative endotracheal aspirate was performed in patients with suspicion of VAT or VAP. Moreover, quantitative endotracheal aspirate was performed in all included patients at the day of random assignment (before starting antibiotics in the antibiotic group) and day 8 after random assignment if patients were still intubated. Microbiological data were available to physicians in different centers. Routine screening of MDR bacteria was performed in study patients at random assignment and weekly thereafter. This screening included nasal and anal swabs. Other microbiologic cultures were performed according to clinical status. In all participating ICUs, weaning from mechanical ventilation was performed according to recommendations of the French Society of Critical Care [17]. The ventilator circuit was not changed routinely. Patients were kept in a semirecumbent position during most of the period of mechanical ventilation. Subglottic secretion drainage and closed tracheal suction devices were not used. No patient received aerosolized antibiotics. All patients were followed until ICU discharge or 28 days after random assignment if they were discharged from the ICU before.

### Definitions

VAT was defined using all of the following criteria [1]: fever (>38°C) with no other recognizable cause, purulent sputum production, positive (≥10^6 ^colony-forming units [cfu] per milliliter) endotracheal aspirate culture [18] yielding a new bacteria (not present at intubation), and no radiographic signs of new pneumonia. All of these criteria had to be present before random assignment. The absence of radiographic signs of new pneumonia was based on physician staff decision in different centers. Only first episodes of VAT occurring more than 48 hours after starting mechanical ventilation were taken into account. VAP was defined by the presence of new or progressive radiographic infiltrate associated with two of the following criteria: (a) temperature of greater than 38.5°C or less than 36.5°C, (b) leukocyte count of greater than 10,000/μL or less than 1,500/μL, and (c) purulent endotracheal aspirate and positive (≥ 10^6 ^cfu/mL) endotracheal aspirate. VAP episodes occurring less than 5 days after starting mechanical ventilation were considered as early-onset. Late-onset VAP was defined as VAP diagnosed at least 5 days after starting mechanical ventilation. Other definitions of nosocomial infections were based on criteria of the Centers for Disease Control and Prevention [19]. Colonization was defined as a positive microbiologic culture without clinical signs of infection. Infection and colonization were considered as ICU-acquired if they were diagnosed more than 48 hours after ICU admission. MDR bacteria were defined as methicillin-resistant *Staphylococcus aureus*, ceftazidime- or imipenem-resistant *Pseudomonas aeruginosa*, *Acinetobacter baumannii*, extending-spectrum β-lactamase-producing Gram-negative bacilli, and *Stenotrophomonas maltophilia*.

Prior antibiotic treatment was defined as any antibiotic treatment during the two weeks preceding ICU admission. In the antibiotic group, antimicrobial therapy was considered appropriate when at least one antibiotic active *in vitro *on all organisms causing VAT was administrated to treat VAT. De-escalation was defined as changing the focus from multiple agents to a single agent if *P. aeruginosa *was not present or as changing from a broad to a narrow agent based on culture data [20]. Severe immunosuppression was defined by the presence of neutropenia (leucocyte count of less than 1,000/μL or neutrophil count of less than 500/μL), active solid or hematology malignancy, long-term corticosteroid therapy (≥1 mg/kg per day for more than 1 month), or HIV infection (CD4 of less than 50/μL during the previous 6 months). COPD was defined according to recent ATS/European Respiratory Society criteria [21]. Impossible-to-wean patients were defined as those patients transferred from the ICU under mechanical ventilation through a tracheostomy tube. The number of mechanical ventilation-free days at 28 days after random assignment was calculated [22]. For example, a patient who survived 28 days and received mechanical ventilation for 10 days was assigned a value of 18. If mechanical ventilation had been used for 10 days and the patient died on day 14, a value of 4 was assigned. The primary endpoint was duration of mechanical ventilation. Secondary endpoints included mechanical ventilation-free days, length of ICU stay, subsequent VAP, ICU mortality, and infection or colonization related to MDR bacteria.

### Statistical methods

SPSS software (SPSS Inc., Chicago, IL, USA) was used for data analysis. Qualitative variables were compared using the chi-square test or the Fisher exact test where appropriate. The distribution of continuous variables was tested. The Student *t *test and the Mann-Whitney *U *test were used to compare continuous variables normally and abnormally distributed, respectively. Results are presented as number (percentage) for frequencies. The results of continuous variables are presented as mean ± standard deviation if normally distributed or as median (interquartile range) for abnormally distributed variables. Odds ratios and 95% confidence intervals were calculated for all significant (*P *< 0.05) qualitative variables. All *P *values were two-tailed. The time to occurrence of ICU death was analyzed in the antibiotic and no antibiotic groups by Kaplan-Meier survival curves.

All analyses were performed on an intention-to-treat (ITT) basis. In addition, a modified ITT analysis was performed after exclusion of (a) patients randomly assigned to the no antibiotic group but who received (for infections other than VAT or subsequent VAP) an antibiotic active *in vitro *on microorganisms responsible for VAT, (b) impossible-to-wean patients, and (c) patients with do-not-resuscitate orders. The aim of this modified ITT analysis was to adjust for these potential confounders.

Based on our previous study [1], it was expected that the duration of mechanical ventilation would be 22 ± 15 days in patients with VAT. The inclusion of 350 patients (175 in each group) was required to detect a difference in mechanical ventilation duration of 5 days between the antibiotic and no antibiotic groups (two-sided α = 0.025, power = 0.90). An interim analysis was planned at the inclusion of 175 patients or 2 years after starting the study if the number of included patients was less than 175.

## Results

**Figure 1:**
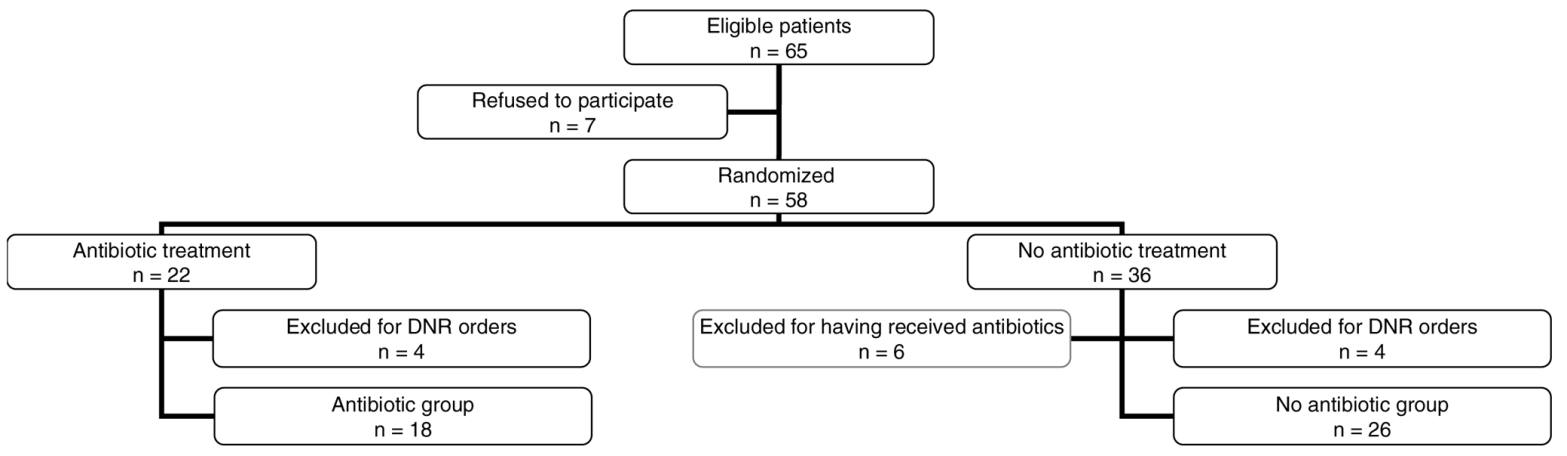
Profile of modified intention-to-treat analysis. DNR, do not resuscitate.

Sixty-five patients were eligible for this study. Seven patients refused to participate. Fifty-eight patients were randomly assigned, including 22 patients in the antibiotic group and 36 patients in the no antibiotic group. Fourteen patients were excluded from the modified ITT analysis (4 of 22 [18%] versus 10 of 36 [27%], *P *= 0.533, in the antibiotic and no antibiotic groups, respectively). Among the 14 excluded patients, 8 patients were excluded for do-not-resuscitate orders (4 of 22 [18%] versus 4 of 36 [11%], *P *= 0.462) and 6 patients were excluded because they were randomly assigned to the no antibiotic group but received antibiotics for infections other than VAT or subsequent VAP (5 bacteremia and 1 severe sepsis) during the 8 days following random assignment. No patient was excluded for impossible weaning from mechanical ventilation (Figure 1).

The planned interim analysis was performed 2 years after starting the study because the number of included patients was less than 175. The study was stopped by the local institutional review board and safety committee because the interim analysis found a significant difference in ICU mortality.

Patient characteristics were similar at ICU admission and at the day of random assignment (Tables 1 and 2). Patients with community-acquired pneumonia at ICU admission had all completed antibiotic treatment for community-acquired pneumonia before inclusion in the study.

**Table 1:** Patient characteristics at intensive care unit admission

	Intention to treat			Modified intention to treat		
	Antibiotic treatment n = 22	No antibiotic treatment n = 36	*P *value	Antibiotic treatment n = 18	No antibiotic treatment n = 26	*P *value
Age, years	62 ± 15	67 ± 12	0.194	61 ± 15	67 ± 12	0.321
Male gender	15 (68)	24 (66)	>0.999	13 (72)	16 (61)	0.531
SAPS II	47 ± 14	47 ± 18	0.994	45 ± 17	48 ± 15	0.481
LOD score	6.6 ± 3.5	6.2 ± 3.6	0.711	6.5 ± 3.8	6.4 ± 3.9	0.990
McCabe			0.687			0.625
Nonfatal underlying disease	10 (45)	14 (38)		10 (55)	11 (42)	
Ultimately fatal underlying disease	9 (40)	17 (47)		7 (38)	12 (46)	
Rapidly fatal underlying disease	3 (13)	5 (13)		1 (5)	3 (11)	
Admission category			>0.999			0.409
Medical	19 (86)	30 (83)		15 (83)	21 (80)	
Surgical	3 (13)	5 (13)		3 (16)	4 (15)	
Trauma	0 (0)	1 (2)		0 (0)	1 (3)	
Comorbidities						
COPD	9 (40)	17 (47)	0.787	7 (38)	12 (46)	0.760
Cardiac failure	6 (27)	4 (11)	0.156	6 (33)	3 (11)	0.128
Cirrhosis	0 (0)	3 (8)	0.281	0 (0)	3 (11)	0.258
Chronic dialysis	4 (18)	2 (5)	0.187	3 (16)	1 (3)	0.289
Diabetes mellitus	6 (27)	3 (8)	0.070	5 (27)	3 (11)	0.204
Transfer from other wards	12 (54)	12 (33)	0.285	9 (50)	9 (34)	0.361
Prior antibiotic treatment	9 (40)	12 (33)	0.585	8 (44)	8 (30)	0.525
Infection at ICU admission	18 (81)	25 (69)	0.365	14 (77)	20 (76)	>0.999
Cause for ICU admission						
Community-acquired pneumonia	6 (27)	10 (27)	>0.999	6 (33)	6 (23)	0.506
Acute exacerbation of COPD	3 (13)	14 (38)	0.073	3 (16)	9 (34)	0.303
Congestive heart failure	3 (13)	1 (2)	0.319	3 (16)	1 (3)	0.289
Neurologic failure	2 (9)	5 (13)	0.698	1 (5)	4 (15)	0.634
Acute poisoning	2 (9)	2 (5)	0.681	2 (11)	2 (7)	>0.999
Others	6 (27)	4 (11)	0.156	3 (16)	4 (15)	>0.999

Results of univariate analysis are presented. Data are expressed as frequency (percentage) or mean ± standard deviation. COPD, chronic obstructive pulmonary disease; ICU, intensive care unit; LOD, logistic organ dysfunction; SAPS, Simplified Acute Physiology Score.

**Table 2:** Patient characteristics at the day of random assignment

	Intention to treat			Modified intention to treat		
	Antibiotic treatment n = 22	No antibiotic treatment n = 36	*P *value	Antibiotic treatment n = 18	No antibiotic treatment n = 26	*P *value
Duration of mechanical ventilation before random assignment, days	17 ± 9	13 ± 6	0.232	17 ± 10	12 ± 6	0.113
SAPS II	33 ± 13	36 ± 13	0.195	32 ± 10	36 ± 12	0.120
LOD score	4.1 ± 2	4.9 ± 2.4	0.185	3.8 ± 1.5	4.8 ± 2.6	0.210
Temperature, °C	38.1 ± 0.6	38.3 ± 0.6	0.408	38.2 ± 0.5	38.2 ± 0.4	0.402
Leucocytes, × 10^9 ^cells/L	12 ± 5.9	12 ± 6	0.619	11.2 ± 4.2	11.9 ± 5.7	0.775
C-reactive protein, mg/mL	111 ± 61	104 ± 80	0.417	104 ± 50	95 ± 67	0.295
Procalcitonin, ng/mL, median (IR)	0.6 (0.10–3.1)	0.8 (0.5–2.7)	0.282	0.7 (0.05–2.8)	0.83 (0.36–2.1)	0.494

Results of univariate analysis are presented. Data are expressed as mean ± standard deviation unless otherwise indicated. IR, interquartile range; LOD, logistic organ dysfunction; SAPS, Simplified Acute Physiology Score.

### Microbiologic results and antimicrobial treatment

**Figure 2:**
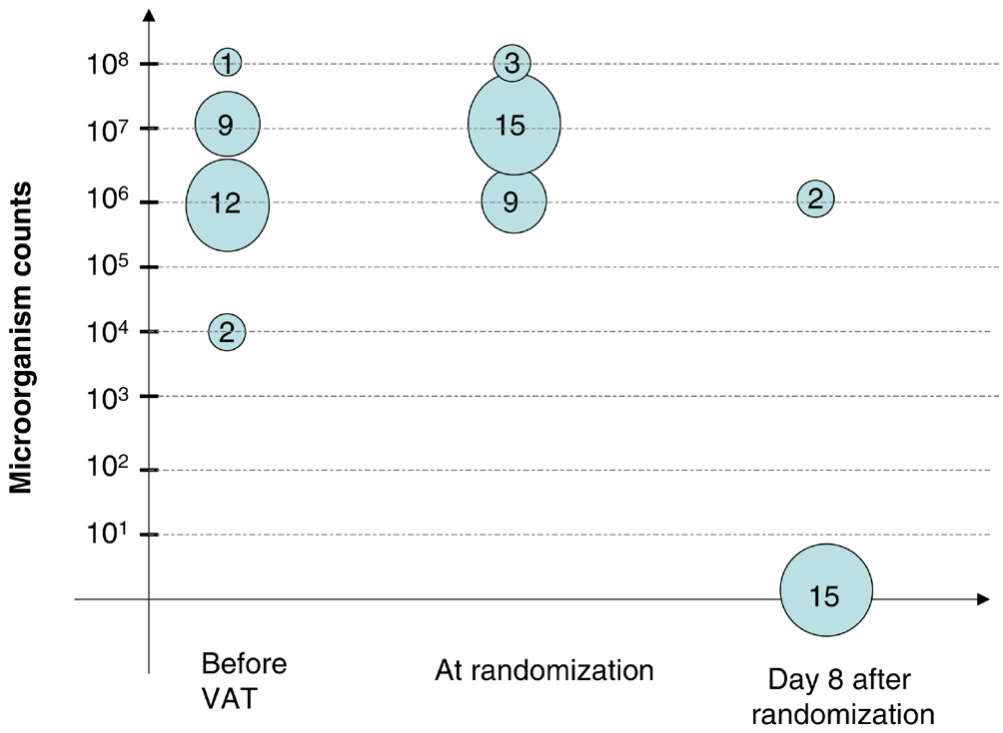
Number of patients randomly assigned to the antibiotic group with different concentrations of microorganisms in the endotracheal aspirate at different time points. Five patients had polymicrobial ventilator-associated tracheobronchitis (VAT).

**Figure 3:**
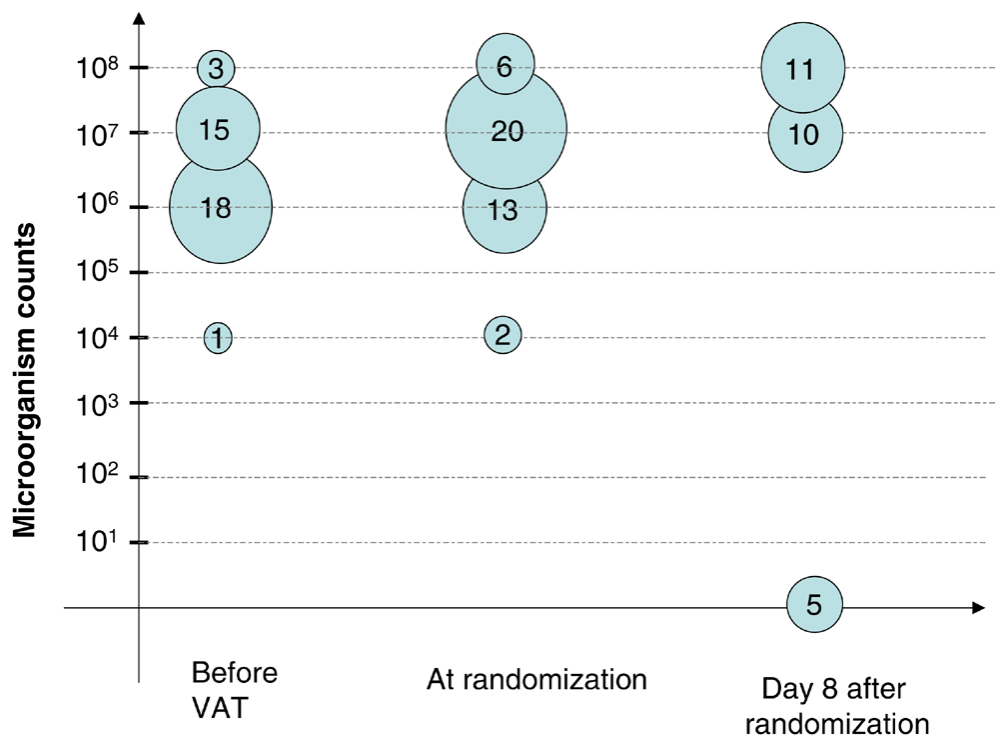
Number of patients randomly assigned to the control group with different concentrations of microorganisms in the endotracheal aspirate at different time points. Two patients had polymicrobial ventilator-associated tracheobronchitis (VAT).

*P. aeruginosa *was the most frequently isolated bacteria in VAT patients (32%). The rate of fluoroquinolone-resistant *P. aeruginosa *was similar in the two groups (6 of 8 [75%] versus 8 of 11 [72%], *P *= 0.689, in the antibiotic and no antibiotic groups, respectively). The microorganisms isolated at a significant threshold are presented in Table 3. In the no antibiotic group, two patients had additional microorganisms cultured at less than 10^6 ^cfu/mL (*P. aeruginosa *and methicillin-sensitive *S. aureus*). The bacteria identified on quantitative endotracheal aspirate at random assignment were the same as those identified on previous endotracheal aspirate in 48 of 58 (82%) patients (17 of 22 [77%] versus 31 of 36 [86%], *P *= 0.481, in the antibiotic and no antibiotic groups, respectively). The number of patients with different concentrations of microorganisms at different endpoints is presented in Figures 2 and 3.

**Table 3:** Bacteria associated with ventilator-associated tracheobronchitis episodes

	Intention to treat		Modified intention to treat	
	Antibiotic treatment n = 22	No antibiotic treatment n = 36	Antibiotic treatment n = 18	No antibiotic treatment n = 26
Microorganisms, number	27	39	22	29
Polymicrobial VAT	5 (22)	3 (8)	4 (22)	3 (11)
MDR bacteria	10 (45)	17 (47)	9 (50)	14 (53)
Gram-negative	20 (90)	27 (75)	16 (88)	20 (76)
*Pseudomonas aeruginosa*	8 (36)	11 (30)	7 (31)	9 (34)
*Enterobacter *species	2 (9)	3 (8)	2 (11)	3 (11)
*Escherichia coli*	3 (13)	3 (8)	1 (5)	2 (7)
*Proteus mirabilis*	3 (13)	1 (2)	2 (11)	1 (3)
*Citrobacter freundii*	1 (4)	2 (5)	1 (5)	1 (3)
*Acinetobacter baumannii*	0 (0)	2 (5)	0 (0)	2 (7)
*Morganella morgani*	1 (4)	2 (5)	1 (5)	1 (3)
*Hemophilus influenzae*	0 (0)	1 (2)	0 (0)	1 (3)
*Stenotrophomonas maltophilia*	1 (4)	1 (2)	1 (5)	0 (0)
*Klebsiella oxytoca*	1 (4)	1 (2)	1 (5)	0 (0)
Gram-positive	7 (31)	12 (33)	6 (33)	9 (34)
Methicillin-resistant *Staphylococcus aureus*	3 (13)	6 (16)	3 (16)	5 (19)
Methicillin-sensitive *S. aureus*	3 (13)	4 (11)	2 (11)	4 (15)
*Streptococcus pneumoniae*	1 (4)	2 (5)	1 (5)	0 (0)

*P *> 0.2 for all comparisons (antibiotic versus no antibiotic treatment). Results are presented as number (percentage) unless otherwise indicated. MDR, multidrug-resistant; VAT, ventilator-associated tracheobronchitis.

In the antibiotic group, 16 of 22 (72%) patients received combination therapy and 6 (27%) patients received monotherapy. Aminoglycosides (45%) and imipenem (40%) were the most frequently prescribed antibiotics (Table 4). In the antibiotic group, 21 of 22 (95%) patients received appropriate initial antibiotic treatment. In the patient who received inappropriate initial treatment, antimicrobial therapy was modified after receipt of identification of causal bacteria (48 hours after random assignment). De-escalation was performed in 4 of 22 (18%) patients.

**Table 4:** Antibiotics prescribed for ventilator-associated tracheobronchitis episodes

	n = 22
Aminoglycosides	10 (45)
Imipenem	9 (40)
Ciprofloxacin	5 (22)
Piperacillin/tazobactam	5 (22)
Ceftazidime	1 (4)
Ticarcillin/clavulanate	1 (4)
Amoxicillin/clavulanate	1 (4)
Methicillin	1 (4)
Ceftriaxone	1 (4)
Vancomycin	1 (4)
Rifampin	1 (4)
Colimycin	1 (4)
Tigecycline	1 (4)
Combination therapy	16 (72)
β-lactams and aminoglycosides	9 (40)
β-lactams and ciprofloxacin	5 (22)
Vancomycin and aminoglycoside	1 (4)
Colimycin and rifampin	1 (4)

Results are presented as number (percentage). Monotherapy was given to patients with ventilator-associated tracheobronchitis related to methicillin-sensitive *Staphylococcus aureus *(n = 2), *Escherichia coli *(n = 2), *Streptococcus pneumoniae *(n = 1), and methicillin-resistant *S. aureus *(n = 1).

### Ventilator-associated pneumonia patients

Twenty of 58 (34%) patients developed subsequent VAP. All VAP episodes were late-onset. Twenty-six microorganisms were identified at a significant threshold in patients with VAP. *P. aeruginosa *was the most frequently isolated bacteria (51%). The rate of VAP episodes related to the same microorganism identified as a causative agent for VAT was significantly lower in the antibiotic group than in the no antibiotic group (0 of 3 [0%] versus 14 of 17 [82%], *P *= 0.018, respectively). No significant difference was found in the duration of mechanical ventilation between random assignment and VAP occurrence (9 ± 6 versus 6.2 ± 4 days, *P *= 0.262, in the antibiotic and no antibiotic groups, respectively). In the control group, no significant difference was found in procalcitonin level at random assignment between patients with subsequent VAP and patients without subsequent VAP (median 0.8 [interquartile range 0.5 to 2.8] versus 0.75 [0.45 to 2.5] ng/mL, *P *= 0.568). Other patient characteristics were also similar in these two subgroups at ICU admission and at random assignment (data not shown).

### Patient characteristics during the intensive care unit stay

Patient characteristics during the ICU stay were similar in the two groups (Table 5). At day 8 after random assignment, the rate of positive endotracheal aspirate was significantly lower in the antibiotic group than in the no antibiotic group (2 of 17 [11%] versus 21 of 26 [80%], *P *< 0.001, respectively).

**Table 5:** Patient characteristics during the intensive care unit stay

	Intention to treat			Modified intention to treat		
	Antibiotic treatment n = 22	No antibiotic treatment n = 36	*P *value	Antibiotic treatment n = 18	No antibiotic treatment n = 26	*P *value
Tracheostomy	5 (22)	5 (13)	>0.999	5 (27)	5 (19)	0.716
Corticosteroid use	8 (36)	19 (52)	0.283	6 (33)	13 (50)	0.359
Septic shock	1 (4)	7 (19)	0.134	1 (5)	5 (19)	0.375
ICU-acquired infections other than VAT and VAP^a^	7 (31)	18 (50)	0.274	6 (33)	13 (50)	0.359
Bacteremia	6 (27)	13 (36)	0.572	5 (27)	8 (30)	>0.999
Urinary tract infection	2 (9)	5 (13)	0.698	2 (11)	5 (19)	0.682
Others	2 (9)	2 (5)	>0.999	2 (11)	2 (7)	>0.999
Total duration of antibiotic treatment, days	25 ± 14	19 ± 15	0.149	24 ± 15	17 ± 15	0.104
Antibiotic treatment before VAT	18 (81)	29 (80)	>0.999	15 (83)	23 (88)	0.676
Antibiotic treatment during the 8 days following random assignment	22 (100)	21 (58)	<0.001	18 (100)	10 (38)	<0.001
Reasons for antibiotic treatment during the 8 days following random assignment						
VAT	22 (100)	0 (0)	<0.001	18 (100)	0 (0)	<0.001
VAP	0 (0)	15 (41)	<0.001	0 (0)	10 (38)	0.003
Other infections	0 (0)	6 (16)	0.073	0 (0)	0 (0)	NA
Antibiotic treatment after day 8 post-random assignment	6 (27)	8 (22)	0.756	3 (16)	5 (19)	>0.999

Results of univariate analysis are presented. Data are expressed as frequency (percentage) or mean ± standard deviation. ^a^Some patients had more than one ICU-acquired infection. ICU, intensive care unit; NA, not applicable; VAP, ventilator-associated pneumonia; VAT, ventilator-associated tracheobronchitis.

### Outcomes

**Figure 4:**
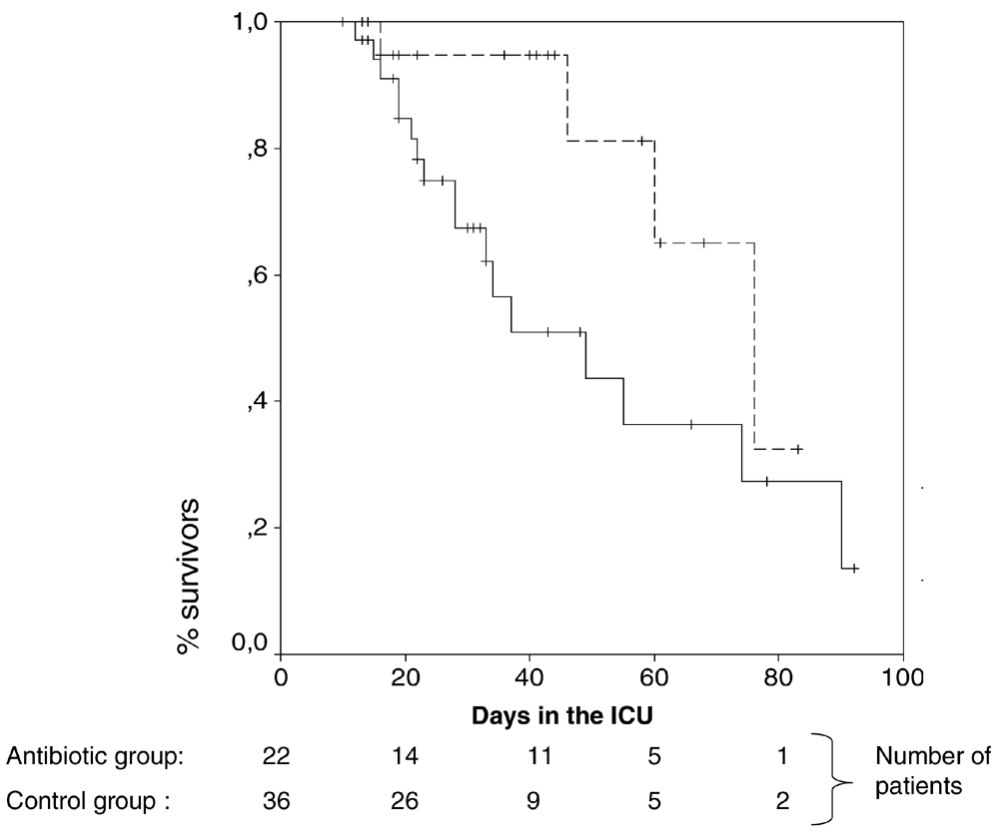
Kaplan-Meier survival curves for patients randomly assigned to the antibiotic and control groups. The dashed line represents the cumulative survival for patients randomly assigned to the antibiotic group, the solid line represents the cumulative survival for patients randomly assigned to the no antibiotic group, and + represents censored patients. *P *= 0.047 by the log rank test. ICU, intensive care unit.

Although the duration of mechanical ventilation and length of ICU stay were similar in the two groups, mechanical ventilation-free days were significantly higher in patients who received antibiotics than in those who did not receive antibiotics. In addition, subsequent VAP and ICU mortality rates were significantly lower in the antibiotic group than in the no antibiotic group. Kaplan-Meier survival curves are presented in Figure 4. Reasons for death included life support withdrawal in 8 patients (4 of 22 [18%] versus 4 of 36 [11%], *P *= 0.462) and multiple organ failure in 13 patients (0 of 22 versus 13 of 36 [36%], *P *< 0.001, in the antibiotic and no antibiotic groups, respectively). No significant difference was found in the rates of infection or colonization related to MDR bacteria diagnosed after random assignment (Table 6). No significant difference was found in outcome between different study centers (data not shown). No *Clostridium difficile *colitis was diagnosed in study patients.

**Table 6:** Outcomes of study patients

	Intention to treat			Modified intention to treat		
	Antibiotic treatment n = 22	No antibiotic treatment n = 36	*P *value	Antibiotic treatment n = 18	No antibiotic treatment n = 26	*P *value
Duration of mechanical ventilation, days	29 ± 17	26 ± 15	0.816	26 ± 15	24 ± 15	0.952
Mechanical ventilation-free days, median (interquartile range)	12 (8–24)	2 (0–6)	<0.001	16 (9–25)	4 (2–10)	0.001
Length of ICU stay, days	40 ± 23	36 ± 21	0.558	37 ± 21	33 ± 20	0.445
Ventilator-associated pneumonia	3 (13)	17 (47)	0.011^a^	2 (11)	12 (46)	0.021^a^
ICU mortality^b^	4 (18)	17 (47)	0.047^a^	0 (0)	11 (42)	0.001^a^
Infection or colonization related to MDR bacteria	9 (40)	13 (36)	0.784	7 (38)	8 (30)	0.748
Ceftazidime or imipenem-resistant *Pseudomonas aeruginosa*	3 (13)	6 (16)	0.534	3 (16)	5 (19)	>0.999
*Acinetobacter baumannii*	0 (0)	2 (5)	0.521	0 (0)	0 (0)	NA
*Stenotrophomonas maltophilia*	1 (4)	0 (0)	0.379	1 (5)	0 (0)	0.409
Methicillin-resistant *Staphylococcus aureus*	2 (9)	1 (2)	0.551	1 (5)	0 (0)	0.409
ESBL-producing Gram-negative bacilli	3 (13)	4 (11)	0.540	2 (11)	3 (11)	>0.999

Results of univariate analysis are presented. Data are expressed as frequency (percentage) or mean ± standard deviation unless otherwise indicated. ^a^Odds ratios (95% confidence intervals) are 0.17 (0.04 to 0.70), 0.14 (0.02 to 0.76), 0.24 (0.07 to 0.88), and 0.45 (0.31 to 0.66), respectively. ^b^Predicted mortality rates, based on Simplified Acute Physiology Score II at ICU admission, were 39%, 39%, 35%, and 41% in the four groups, respectively. ESBL, extended-spectrum β-lactamase; ICU, intensive care unit; MDR, multidrug-resistant; NA, not applicable.

## Discussion

The main results of our study are the following: (a) In patients with VAT, antibiotic treatment was associated with significantly lower ICU mortality and subsequent VAP rates and more mechanical ventilation-free days. (b) No significant difference was found in the rate of infection or colonization related to MDR bacteria diagnosed after random assignment between the two groups. (c) No significant difference was found in the total duration of mechanical ventilation or ICU stay between the antibiotic and no antibiotic groups.

To our knowledge, this is the first randomized study aiming at evaluating the impact of antibiotic treatment on the outcome of patients with VAT. The beneficial effect of antibiotics found in this study on the number of days free of mechanical ventilation could be explained by the reduction of secretion volume and tracheobronchial inflammation. Palmer and colleagues [23,24] investigated the impact of aerosolized antibiotics on secretion volume in chronically mechanically ventilated patients with VAT. In those studies, aerosolized antibiotics eradicated respiratory pathogens, decreased inflammatory cells and the volume of secretions, and were not associated with increased resistance. Increased secretion volume is a well-known risk factor for difficult weaning from mechanical ventilation [25]. However, these factors were not evaluated in our study. The absence of a significant difference in the total duration of mechanical ventilation is probably related to the small number of patients included in the study as compared with the number of patients required to demonstrate a significant difference. However, the number of days free of mechanical ventilation was significantly higher in the antibiotic group. This could be explained by the fact that the mortality rate was significantly higher in patients in the no antibiotic group and by the longer duration of mechanical ventilation before random assignment in the antibiotic group.

Lower rates of VAP and ICU mortality were found in VAT patients who received antimicrobial treatment. Similar results were found in a recent randomized study conducted in COPD patients mechanically ventilated for severe acute exacerbation [26]. However, in that study, all included patients had community-acquired bronchitis. In addition, no bacteria could be found in 38% of included patients. Although the severity of illness and predicted mortality were similar in the two groups, mortality rate was significantly higher in the control group. This result is probably related not to VAT but to the higher rate of VAP in control patients. In addition, all VAP episodes were late-onset and the rate of *P. aeruginosa *VAP was high. Previous studies demonstrated that VAP was associated with increased mortality rate [27,28]. A recent study found higher mortality rates in patients with late-onset VAP as compared with patients with early-onset VAP [28]. *P. aeruginosa *VAP was also found to be associated with high mortality rates [29]. However, other studies suggested that VAP was not associated with an increased mortality rate [30,31]. Another potential explanation for the higher mortality rate in untreated patients is the possible presence of pneumonia in these patients. VAT may be difficult to differentiate from VAP because of the low sensitivity of chest portable radiographs in ICU patients [32,33]. Though not statistically significant, the duration of mechanical ventilation from random assignment to VAP occurrence was shorter in the no antibiotic group than in the antibiotic group. This result suggests that VAP might have been present at the time of random assignment despite the absence of new infiltrate on the chest radiograph. In a prospective, observational, multicenter, cohort study performed on 2,706 patients, outcomes of patients with suspected pneumonia and normal chest radiographs (33%) have been prospectively investigated [34]. Similar rates of positive sputum cultures, positive blood cultures, and mortality were found in patients without radiographic pneumonia as compared with patients with radiographic pneumonia. In a recent study [35], accuracy of chest radiography was compared with high-resolution computed tomography (HRCT) in 47 patients with suspected community-acquired pneumonia. HRCT identified all 18 community-acquired pneumonia cases (38%) apparent on radiographs as well as 8 additional cases (17%). The performance of HRCT could be suggested to better diagnose VAP in critically ill patients. However, recent guidelines require the presence of new infiltrate on a chest radiograph as a criterion for VAP diagnosis [15]. Therefore, a baseline examination should be available for all patients to diagnose a new infiltrate on HRCT. Such a strategy would be expensive and difficult to apply in critically ill patients. The absence of new infiltrate on a chest radiograph could be more difficult to diagnose in patients with an abnormal chest radiograph at ICU admission. In our study, 38% of study patients had an abnormal chest radiograph at ICU admission. However, patients admitted to the ICU frequently have an abnormal chest radiograph [36].

The rate of COPD (44%) was high. However, no significant difference was found in COPD rate between the two groups. A previous observational study identified COPD as a risk factor for VAT [1]. The rate of patients with multiple organ failure was significantly higher in the control group than in the antibiotic group. This result could be explained by the higher rate of VAP in these patients. Previous studies found VAP to be associated with multiple organ failure [37,38].

VAT could also be difficult to differentiate from lower respiratory tract colonization. Several factors support the presence of infection rather than colonization in our patients: (a) Quantitative endotracheal aspirate was used with a high threshold (10^6 ^cfu/mL) to diagnose VAT, (b) only new bacteria were taken into account, (c) all patients had fever, and (d) leucocyte, C-reactive protein, and procalcitonin levels were high in study patients. Although fever and high leucocyte and C-reactive protein levels may simply reflect the presence of systemic inflammatory response, procalcitonin is useful in differentiating bacterial sepsis from systemic inflammatory response in critically ill patients [39-41]. However, the exclusion of pathogens present at the time of intubation could be a matter of debate since these pathogens could be responsible for VAT. In addition, microorganisms cultured at a lower concentration (<10^6 ^cfu/mL) might be associated with VAT [2]. On the other hand, one could argue that patients with a high microorganism count on tracheal aspirate cultures and no radiographic infiltrates should be treated with antibiotics. However, stable patients receiving prolonged mechanical ventilation without clinical pneumonia have a high alveolar burden of bacteria [42]. Therefore, the presence of purulent tracheal aspirate and fever is important to determine patients who would benefit from antimicrobial treatment.

This study has some limitations. First, the trial stopped early after the planned interim analysis showed a significant reduction of ICU mortality rate in the antibiotic group. Therefore, several random assignment blocks could not be ended, resulting in an imbalance in the numbers of patients randomly assigned to the antibiotic or control group. One could argue that no significant difference was found in the primary endpoint. However, the significant difference in ICU mortality, subsequent VAP, and mechanical ventilation-free days represents overwhelming evidence of benefit to justify stopping the trial early. In addition, this endpoint was no longer relevant given the difference in ICU mortality. Second, the study was not blinded and antibiotic treatment was not standardized in all treated patients. However, blinding was not possible using a targeted antibiotic strategy based on results of previous endotracheal aspirate culture. The aim of such a strategy was to reduce the usage of broad-spectrum antibiotics and to provide a higher rate of appropriate initial antibiotic treatment. A recent study found routine surveillance endotracheal aspirate useful to prescribe appropriate antibiotic therapy in patients with VAP [43]. Furthermore, the results of our study were not evaluated by an independent committee to account for the absence of blinding. However, ICU mortality was significantly different between the two groups. This outcome does not need to be assessed by a committee blinded to patient assignment. Third, the number of patients screened for this study could not be provided and the number of included patients was lower than initially expected. Potential reasons for this slow recruitment include strict inclusion and exclusion criteria and difficulties in differentiating VAT from VAP. Fourth, 21 of 36 (58%) patients randomly assigned to the no antibiotic group had received antibiotics during the 8 days following random assignment, including 15 patients (41%) for subsequent VAP and 6 patients (16%) for infections other than VAT or subsequent VAP. However, subsequent VAP was a secondary endpoint. In addition, to adjust for this confounding factor, we have performed a modified ITT analysis excluding those patients randomly assigned to the no antibiotic group but who received antibiotics for infections other than VAT or subsequent VAP. Fifth, a computed tomography scan was not systematically performed to search for nosocomial sinusitis. In addition, no information could be provided on hospital mortality, oral care, and type of nutrition. Finally, because of the small sample size, a type I error could not be excluded. However, the significant difference found in ICU mortality is probably related to the significant difference in subsequent VAP rates between the two groups.

## Conclusion

We conclude that, in patients with VAT, antimicrobial treatment is associated with a greater number of days free of mechanical ventilation and lower rates of VAP and ICU mortality. However, antibiotic treatment has no significant impact on the total duration of mechanical ventilation or ICU stay.

## Key messages

• In patients with ventilator-associated tracheobronchitis, antibiotic treatment is associated with significantly lower intensive care unit (ICU) mortality and subsequent ventilator-associated pneumonia rates and more mechanical ventilation-free days.

• In these patients, antibiotic treatment has no significant impact on the total duration of mechanical ventilation or ICU stay.

## Abbreviations

ATS = American Thoracic Society; cfu = colony-forming units; COPD = chronic obstructive pulmonary disease; HRCT = high-resolution computed tomography; ICU = intensive care unit; ITT = intention-to-treat; MDR = multidrug-resistant; VAP = ventilator-associated pneumonia; VAT = ventilator-associated tracheobronchitis.

## Competing interests

The authors declare that they have no competing interests.

## Authors' contributions

SN helped to design the study and to collect data, had full access to all data in the study, wrote the manuscript, and had final responsibility for the decision to submit it for publication. DM and AD helped to design the study and to collect data. RF, EJ, F Decamps, F Dewavrin, and GB helped to collect data. CDP performed statistical analyses. All authors participated in critical revision of the manuscript. All authors read and approved the final manuscript.
